# Seasonal spatial heterogeneity of warming rates on the Tibetan Plateau over the past 30 years

**DOI:** 10.1038/srep11725

**Published:** 2015-06-26

**Authors:** Jianping Duan, Lun Li, Yongjie Fang

**Affiliations:** 1 State Key Laboratory of Vegetation and Environmental Change, Institute of Botany, Chinese Academy of Sciences, Beijing, 100039, China; 2 Chinese Academy of Meteorological Sciences, Beijing, 100081, China; 3 National Climate Center, China Meteorological Administration, Beijing 100081, China

## Abstract

Based on temperature data from 79 meteorological stations, we estimate the warming rate by season on the Tibetan Plateau (TP) during 1984–2013. The warming rate was spatially heterogeneous across seasons over the past 30 years. The northern TP (NTP) experienced more warming than the southern TP (STP) (divided near 33°N) in all seasons. The greatest north-south difference in warming was 0.70 ± 0.11 °C for summer (June-August), while the smallest difference was 0.27 ± 0.14 °C for the cold season (November-April). Such seasonal and spatial heterogeneity in the warming rate is consistent with the seasonal precipitation patterns of the NTP and the STP. One possible cause for this phenomenon is that more precipitation occurs in the STP than in the NTP (especially for summer), accompanied by more low cloud cover, which may have slowed the warming rate. Our results imply that dry regions on the TP will possibly experience greater temperature increase than wet regions under future global warming, and this will be more prominent in summer.

The Tibetan Plateau (TP), covering more than 2.5 million km^2^ and with an average elevation of more than 4000 m a.s.l., is the highest and largest plateau in the world. Over recent decades, the TP has experienced persistent and extensive warming^1^,^2^,^3^, and the linear rates of temperature increase for this region have exceeded those of both the Northern Hemisphere and the region’s latitudinal zone^1^,^4^. The TP is one of the areas most sensitive to global climate change^1^,^2^. Climate change in this region has strong effects on both climate and the ecosystems of the Asian continent and even throughout the world^3^,^5^.

Some studies reported that the strongest warming trend on the TP occurred in the winter months for the latter half of the 20th century^5^,^6^,^7^, and the trend is especially prominent in the higher altitudes^1^,^7^. However, Guo and Wang^8^ argued that the increase in summer temperature in the northeastern TP has played a primary role in the rapid warming that has taken place since the mid-1980s and noted that the Qaidam Basin in the northeastern TP, the driest region in the TP, experienced the most significant warming in TP from 1961 to 2007. A better understanding of these results requires further exploration of the seasonal and spatial patterns of warming rates on the TP.

In fact, some climate conditions in the large-scale TP are well known to be spatially different (e.g., summertime moisture differs between the NTP and the south STP)^9^,^10^. Moreover, the observed records show that the most rapid warming on the TP during 1951–2013 occurred in the last three decades (Fig. 1). In this study, we hypothesize that the spatially heterogeneous climate of the TP can create a spatially heterogeneous warming rate. Subsequently, we examine this hypothesis based on the most pronounced warming period, 1984–2013, and explore potential explanations for the results.

## Results

The spatial patterns of warming rates were calculated from the 79 stations for different seasons as shown in Fig. 2. Most of the stations present significant warming trend in each season over the past 30 years (Supplementary Fig.S1 online). Obviously, warming rates in summer and the warm season create spatial heterogeneity between the NTP and the STP (the boundary is near 33°N) (Figs 2a,b). The warming rate is generally more than 0.6 °C/decade for the NTP over the past 30 years, and it is less than 0.4 °C/decade for the STP (Figs 2a,b). The difference in warming amplitude between the NTP and STP (NTP minus STP) is 0.70 ± 0.11 °C for summer and 0.58 ± 0.10 °C for the warm season (Fig. 3). However, warming rates in winter and the cold season do not show strong north-south heterogeneity over the past 30 years, and the highest warming rate occurs in the central TP (Figs 2c, d). The north-south differences of warming amplitude are 0.32 ± 0.20 °C and 0.27 ± 0.14 °C for these two seasons over the past 30 years, respectively (Fig. 3). For spring, autumn and the annual mean, the warming rate in the NTP is generally higher than in the STP (Figs 2e,f and g). The north-south difference in warming amplitude is 0.54 ± 0.13 °C for spring, 0.36 ± 0.13 °C for autumn and 0.40 ± 0.11 °C for the annual mean temperature (Fig. 3). The difference among these three values is less than between summer and the warm season but greater than between winter and the cold season (Fig. 3). Comparison of warming rates between winter and summer (Figs 2a,c) shows that the former is greater than the later in the central TP, but the latter is greater than the former in the northeastern TP. For a specific station, such comparison sometimes depends on the analysis period of climate data (Supplementary Table S2 online). In fact, in the large-scale Northern Hemisphere, asymmetric results were also obtained due to different analyses periods^9^. This may be one of reasons why discrepant results have been reported by different studies. Moreover, a common feature of the warming rate for each season is that the highest warming rate generally appears in the Qaidam Basin region (the driest region in the TP) and the lowest warming rate occurs in the southeastern TP (the wettest region in the TP) (Figs 2a–g).

## Discussion

What created the spatial variation in the TP warming rate over the last three decades? Two features of the warming rate should be considered. One such feature is the difference above and below 33°N, the boundary between the NTP and the STP, which has also been suggested as an important “divider” between the NTP and the STP for other climatic characteristics^10^,^11^. For example, the 33°N is the northern boundary of the Indian monsoon and the northernmost reach of intertropical convergence zone (ITCZ) on the TP in summer^10^. The warming rate also varies through space according to the season (Fig. 2). Summer saw the greatest difference in warming between the NTP and the STP, and the difference was smallest in the cold season (Figs 2a,d and 3). To further explore the reason, we analyzed the spatial precipitation pattern for each season on the TP (Fig. 4). Similar to the spatial warming rate (Fig. 2), the spatial precipitation differed noticeably above and below 33 °N on the TP in summer and the warm season (Figs 4a,b and 3), but there was little difference in winter and the cold season (Figs 4c,d and 3). There was a north-south difference in spring, autumn and in the annual precipitation, but the difference fell between the high summer variation and the low winter variation (Figs 4e,f,g and 3). Moreover, there is no clear north-south boundary between precipitation patterns for the three seasons (Figs 4e,f and g). Thus, the seasonal spatial heterogeneity of warming rate on the TP matches well with the north-south precipitation pattern for each season over the past 30 years (Figs 2, 4 and 3). This phenomenon is further validated by the high warming rate in the dry Qaidam basin and the low warming rate in the wet southeastern TP for each season (Figs 2, 4).

How do the seasonal warming patterns relate to the seasonal precipitation patterns on the TP? Low cloud cover may be responsible. Our analyses show that spatial patterns in low cloud cover (Fig. 5) are also generally consistent with the spatial warming rates for each season (Fig. 2). Low could cover differs most markedly between the NTP and the STP in summer and the warm season (−22.4 ± 3.9% for summer and −20.1 ± 4.1% for the warm season) and differs least in winter and the cold season (−12.8 ± 2.9% for winter and −15.3 ± 3.2% for the cold season) (Figs 5 and 3). A synoptic analysis also demonstrated that summertime convective cloud systems on the TP mainly occurred south of 32 °N^12^. The influence of low clouds on surface temperatures has been suggested by previous studies^13^,^14^,^15^,^16^. Low clouds contain liquid droplets with a high albedo, and the low height and strong cloud-top heating prevent them from absorbing much longwave radiation^13^,^14^,^15^,^16^. As a result, such cloud cover has a cooling effect on the atmosphere and surface below the cloud^13^,^14^,^15^,^16^. Considering the poor coverage of meteorological stations in the western TP, understanding of the results in the region should be with caution. The spatial patterns of precipitation and low cloud cover could be the cause of the spatially heterogeneous warming rate on the TP. However, the cause of warming rate variation with time on the TP is complex and related to several factors^8^,^17^.

## Conclusion

Although global warming experienced a hiatus in the last decade, climate warming on the TP was marked by persistent and extensive temperature increases. The warming rate on the TP shows seasonal spatial heterogeneity between the NTP and the STP (roughly defined as above and below 33°N). Over the period 1984–2013, the greatest difference in warming amplitude between the NTP and STP occurred in summer (June-August) (0.70 ± 0.11 °C) and the warm season (May-October) (0.58 ± 0.10 °C), while there was less difference in winter (December-February) (0.32 ± 0.20 °C) and the cold season (November-April) (0.27 ± 0.14 °C). Such spatial heterogeneity in warming rates between seasons is consistent with their respective precipitation, which was possibly related to the spatial heterogeneity of low cloud cover among seasons on the TP (i.e., a large difference occurs in summer and there is little difference in winter). These results imply that dry regions will possibly experience greater temperature increase than wet regions under future global warming.

## Methods

**Figure 1:**
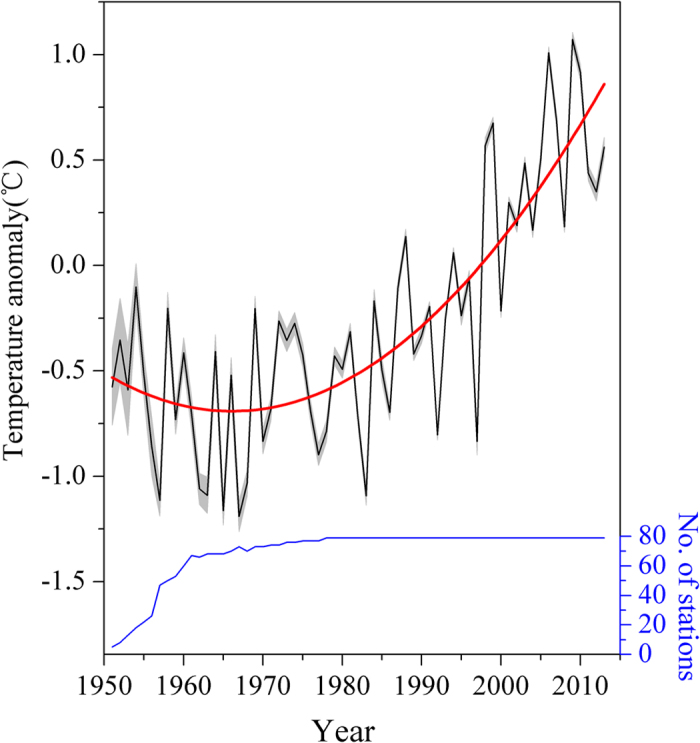
Annual mean temperature anomalies for the TP from 79 meteorological stations (black line) and the number of stations back to time (blue line). The red line is the second-order polynomial fitting of the annual mean temperature anomalies. The shaded area is the error bar.

**Figure 2:**
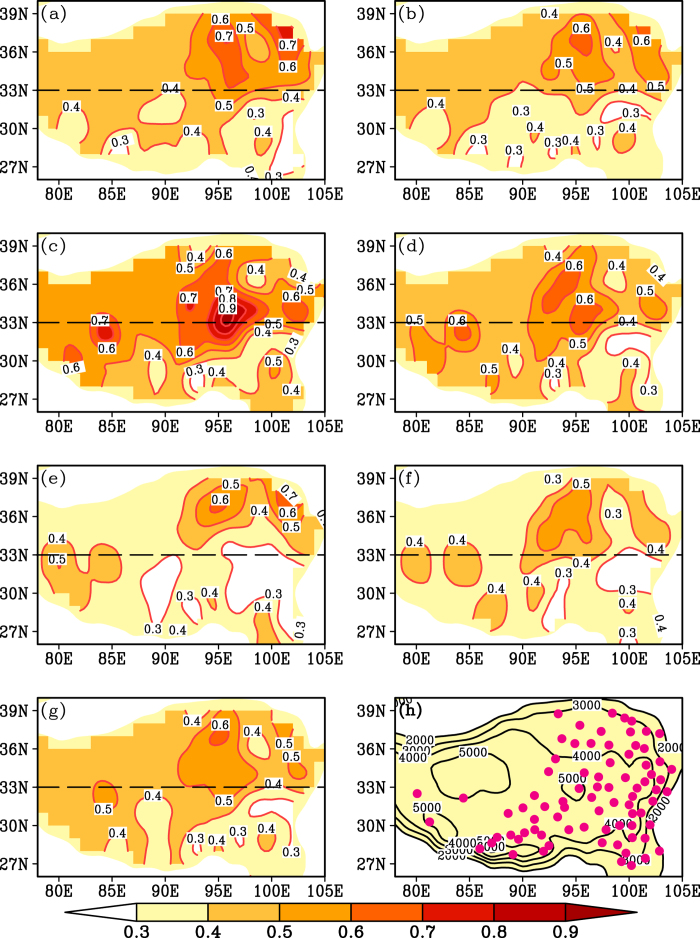
Spatial patterns of warming rate calculated from 79 stations for (a–g) seven annual intervals ranging from 3 to 12 months during 1984–2013 and (h) the location of the 79 stations and the elevation contours of the TP. (**a**) Summer (June-August), (**b**) Warm season (May-October), (**c**) Winter (December-February), (**d**) Cold season (November-April), (**e**) Spring (March-May), (**f**) Autumn (August-October), (**g**) Annual mean (January-December). The unit for contour line is °C/decade and is m for (**h**).

**Figure 3:**
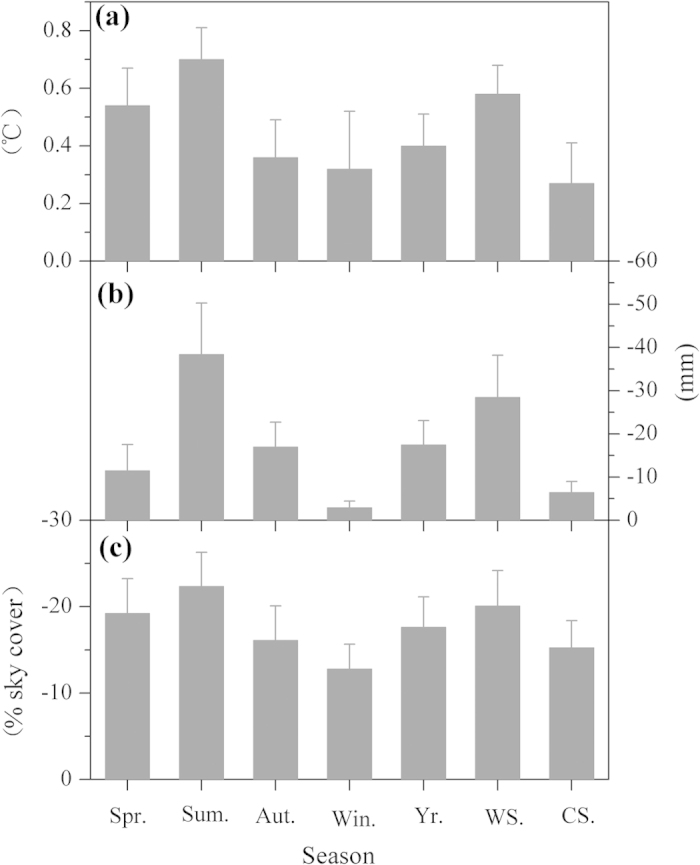
The north-south (above and below 33 °N) differences for (a) warming amplitude, (b) precipitation and (c) low cloud cover for the seven time intervals over the last 30 years (1984–2013). The differences were calculated as the NTP minus the STP. Error bars are presented for each season. Spr., Sum., Aut., Win., Yr., Ws. and CS. represent spring, summer, autumn, winter, annual average, the warm season and the cold season, respectively. The months included in each season are indicated in Fig. 2.

**Figure 4:**
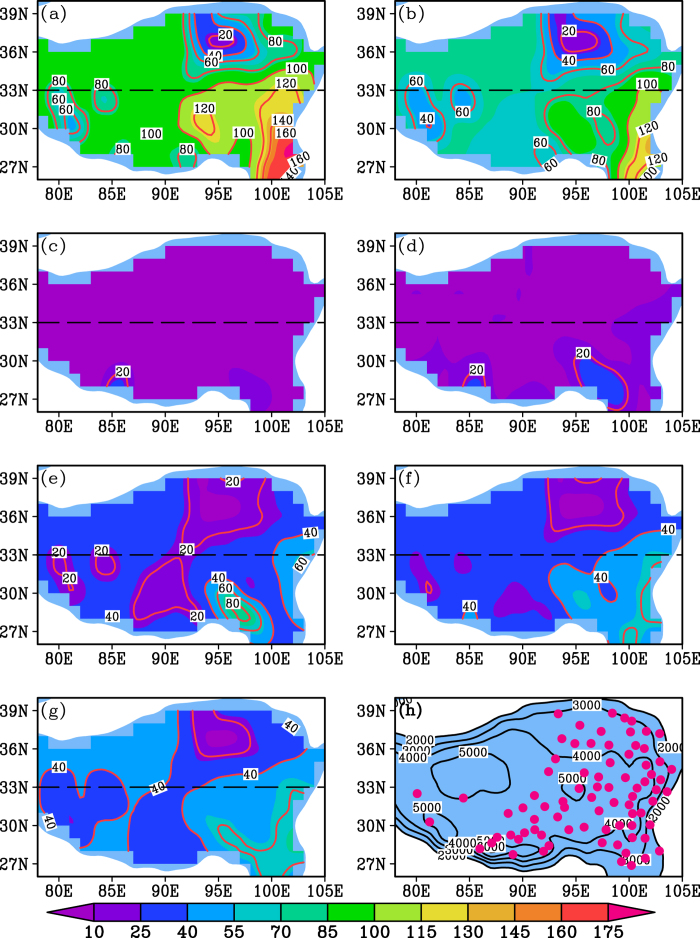
Spatial patterns of monthly mean precipitation for (a–g) seven time intervals calculated from 79 stations during 1984–2013 and (h) the location of the 79 stations and the elevation contours of the TP. (**a**) Summer (June-August), (**b**) Warm season (May-October), (**c**) Winter (December-February), (**d**) Cold season (November-April), (**e**) Spring (March-May), (**f**) Autumn (August-October), (**g**) Annual mean (January-December). The unit for contour line is mm and is m for (**h**).

**Figure 5:**
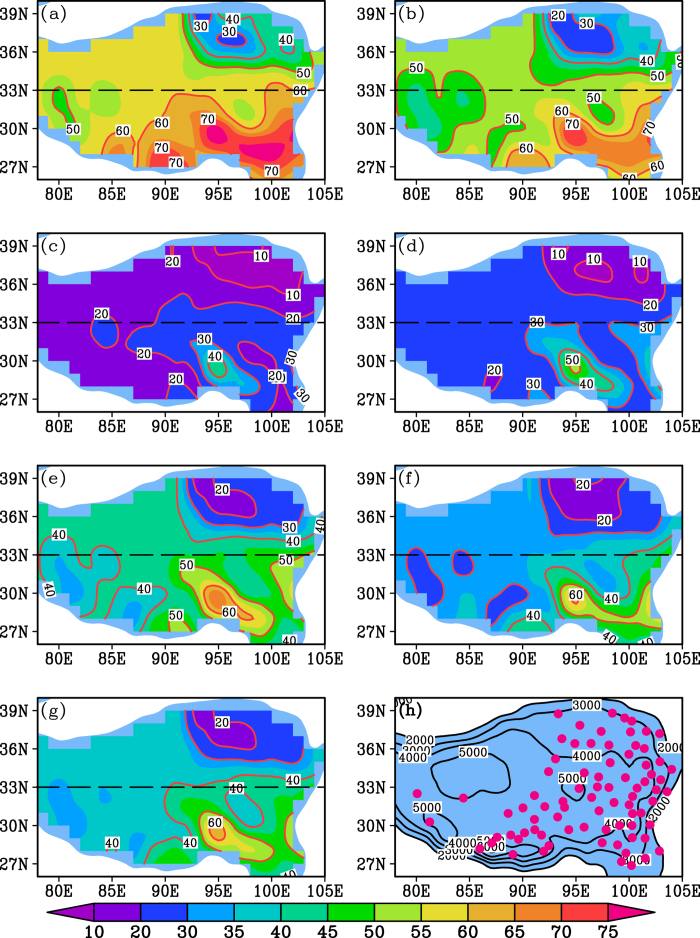
Same as Fig. 4, but for low cloud cover. The unit for contour line is % sky cover and is m for (h).

Monthly climate data from 83 TP meteorological stations at altitudes above 2000 m a.s.l. were obtained from the National Meteorological Information Center of China Meteorological Administration. Preliminary quality control of the climate datasets has been performed by the National Meteorological Information Center of China Meteorological Administration. In this study, we excluded the years lacking data for more than four monthly values, while missing data for less than four monthly values were replaced with values calculated using simple linear interpolation between the two nearest known values (Fig. 1). Over all, there were 58 missing monthly data from 27 stations between 1951 and 2013. All the interpolated values have been validated well and a sample is presented in the Supplemental Information (Supplementary Fig. S2 and Table S1 online). The homogeneity of climate data is assessed using the RHtest software (available from the ETCCDMI website), which uses a two-phase regression model to check for multiple step-change points that could exit in a time series^18^,^19^. After quality control and homogeneity test, data from 79 stations were used in this study (Figs 1 and 2h). The length of records among each station was different, but they all included the last 30 years (i.e., 1984–2013) (Fig. 1). Our units of analysis included seven annual intervals ranging from 3 to 12 months. These were winter (December-February), spring (March-May), summer (June-August), autumn (August-October), the entire cold season (November-April), the entire warm season (May-October), and the annual mean (January-December). The monthly mean temperature data recorded in each station were used to calculate the large-scale annual temperature anomaly on the TP (Fig. 1). Because the most prominent warming trend was between 1984 and 2013 (Fig. 1), and these years had the most available stations and no missing data, the warming rate was calculated for each station during 1984–2013. Data for most stations have a better homogeneity from 1984 to 2013 than the entire period. The monthly total precipitation and monthly low cloud cover data for the same years were used to reveal the climatic context of the warming rate. There was one month with missing data for precipitation and four months with missing data for low cloud cover, which were replaced with values calculated using simple linear interpolation described above.

Warming rate was calculated using the nonparametric approach of Sen’s robust slope estimator based on Kendall’s τ^20^, which has been widely used in climate studies. Uncertainties were calculated as the standard error (SE) of the values from all stations.

## Additional Information

**How to cite this article**: Duan, J. *et al.* Seasonal spatial heterogeneity of warming rates on the Tibetan Plateau over the past 30 years. *Sci. Rep.* **5**, 11725; doi: 10.1038/srep11725 (2015).

## Supplementary Material

Supplementary Information
