# Supplementary material for: Seasonal spatial heterogeneity of warming rates on the Tibetan Plateau over the past 30 years
Source: Sci Rep. 2015 Jun 26;5:11725. doi: 10.1038/srep11725 (PMC4481825; doi:10.1038/srep11725)
Supplement: Supplementary Information [file srep11725-s1.pdf]

Supplementary Information

Seasonal spatial heterogeneity of warming rates on the Tibetan Plateau over the past 30 years

Jianping Duan, Lun Li & Yongjie Fang

Figure S1. Confidence level (*p* value) of warming rates for each station in seven time intervals presented in Figure 2.

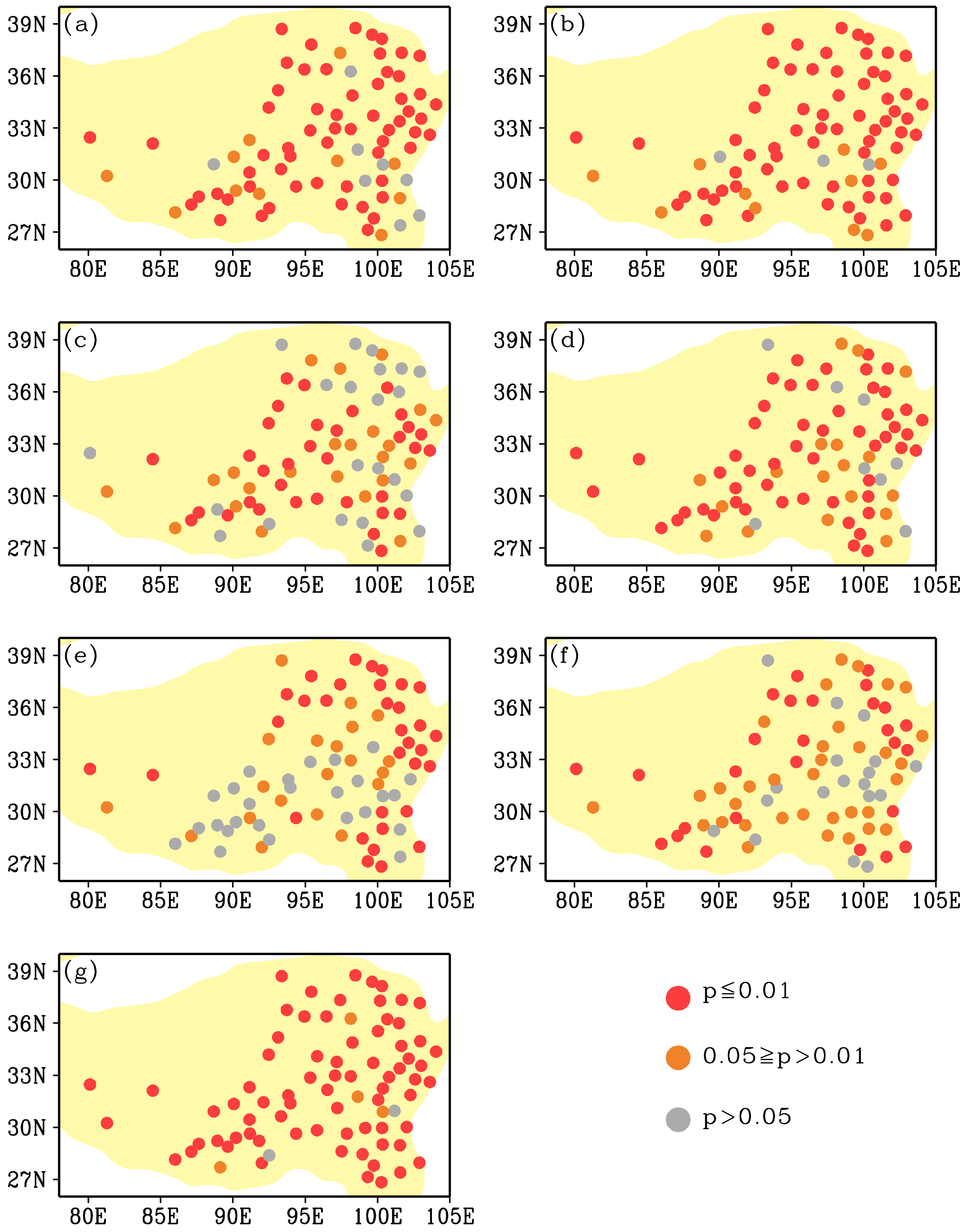

Figure S2. Comparison of the March temperature values between Shenzha station and Shigatse station. The interpolated March temperature value in 1960 for Shenzha station is noted.

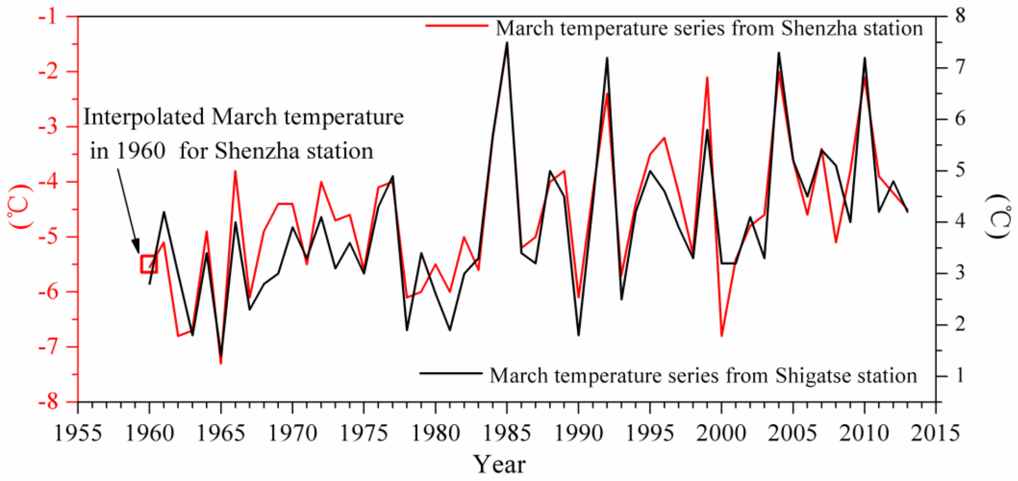

Missing March temperature value in 1960 for Shenzha station was interpolated by linear regression using the March temperature series from the near Shigatse station during 1960-2013. The correlation coefficient between the two March temperature series is 0.9 (R^2=0.81, p<0.0001) during the period 1961-2013. Comparison of the two series shows good homogeneity (**Fig. S2**). The statistics of the regression model are highly significant, and the prediction skill of the regression model can be validated well using cross-validation leave-one-out method. The validation statistics are listed in **Table S1**.

Table S1. Statistics of the calibration and verification for the regression model.

	Calibration				Leave-one-out verification			
Period	r	R^2	R^2_{adj}	F	RE	ST	ST1	PMT
1961–2011	0.90	0.81	0.806	217.2	0.8	46/7	42/10	4.3

RE = reduction error, ST= sign test, ST= first difference sign test, PMT = product mean test. Statistics r, R^2, R^2_{adj}, F, ST, ST1 and PMT are all significant at the level of $p < 0.001$.

Table S2. Warming rate comparison between winter and summer in two periods for 5 meteorological stations located in the northeastern TP.

Station	Latitude/Longitude	1961-2013		1984-2013	
		Winter	Summer	Winter	Summer
Dachaidan	37.85°N/95.37°E	0.76 °C/10yr	0.44°C/10yr	0.69°C/10yr	0.78 °C/10yr
Dulan	36.30°N/98.10 °E	0.40°C/10yr	0.18°C/10yr	0.11°C/10yr	0.31°C/10yr
Lenghu	38.75°N/93.33 °E	0.38 °C/10yr	0.24 °C/10yr	0.19°C/10yr	0.52 °C/10yr
Nuomuhon	36.43°N/96.42 °E	0.51 °C/10yr	0.41 °C/10yr	0.42°C/10yr	0.77 °C/10yr
Gangcha	37.33°N/100.13 °E	0.41 °C/10yr	0.31 °C/10yr	0.31°C/10yr	0.59 °C/10yr

Note: red font indicates greater warming rate.
